# Recent Advances in Lycopene for Food Preservation and Shelf-Life Extension

**DOI:** 10.3390/foods12163121

**Published:** 2023-08-20

**Authors:** Zhixi Li, Fanqianhui Yu

**Affiliations:** 1 Haide College, Ocean University of China, Qingdao 266100, China; lizhiqian@stu.ouc.edu.cn; 2 Department of Computer Science and Technology, Ocean University of China, Qingdao 266100, China; 3 College of Food Science and Engineering, Ocean University of China, Qingdao 266003, China

**Keywords:** lycopene, antioxidant, phytochemical compound, food products

## Abstract

In recent years, there has been increasing concern about the safety of additives used to extend the shelf-life of food products. As a result, lycopene, a natural phytochemical compound, has attracted attention, as it has been demonstrated to be a potential alternative to traditional artificial antioxidants, with significant health benefits when applied to food preservation. Based on this, this review introduces the specific forms of lycopene currently used as an antioxidant in foods, both in its naturally occurring forms in fruits and vegetables and in artificially added forms involving technologies such as composite coating, active film packaging, emulsion, and microcapsules. In addition, it also provides a comprehensive summary of the effects and progress of lycopene in the preservation of different types of food products, such as meat, seafood, oil, dairy products, fruits, and vegetables, in the last decade. At last, it also points out the limitations of lycopene, including its insolubility in water, dark color, and high sensitivity to heat or light, as well as the potential solutions to load lycopene on suitable carriers, such as combining lycopene with antimicrobial substances or other actives, in order to broaden its applications as an antioxidant in future foods.

## 1. Introduction

Food preservation plays an important role in extending the shelf-life of food products and is closely related to their quality and sensory properties [1]. In general, preservatives and antioxidants are used together for food preservation, with preservatives used to prevent spoilage and antioxidants used to delay chemical oxidization processes that produce unpleasant tastes and odors [2]. Specifically, chemical and synthetic preservatives, such as sodium benzoate, potassium sorbate, nitrates, etc., have been widely used in food preservation [3]. They are very cheap and widely used in the food industry, but according to recent studies, long-term consumption of products containing artificial preservatives and additives may cause many physical hazards involving neurotoxicity, accelerated aging, teratogenic, and a range of other effects [3,4]. Due to several health problems mentioned above, people’s awareness of the use of these synthetic antioxidants has gradually increased. The most potent synthetic antioxidant, tert-butylhydroquinone (TBHQ), is not permitted to be used in food in several developed nations like Japan, Canada, and Europe. Butylated hydroxyanisole (BHA) has also been taken off the Generally Recognized as Safe (GRAS) list of compounds [5]. In addition, as the concept of food safety and health becomes more prevalent, consumers pay more attention to the naturalness and safety of products when purchasing them. Therefore, using natural preservatives and antioxidants from plants and other natural sources to extend the shelf-life of foods has become a significant research interest in recent years and can be an effective alternative.

Lycopene has been found to be a good choice in the search for alternatives to synthetic antioxidants. It is a type of phytochemical compound found mainly in tomatoes and is commonly extracted or purified through organic solvents [6]. Some new methods have been recently applied, such as using supercritical carbon dioxide (SC-CO_2_) extraction, combining SC-CO_2_ extraction with microwave irradiation pre-treatment, or even directly producing lycopene from CO_2_ in photoautotrophic bacteria [7,8,9]. In addition to tomatoes, lycopene is also widely distributed in watermelon, papaya, guava, and some other red fruits and vegetables [10]. The amount of lycopene in different plants varies, and can be impacted by factors such as soil quality and growing conditions like temperature. For instance, tomatoes grown outdoors have higher levels of lycopene than those grown indoors [11]. Moreover, tomato is the most widely produced and consumed fruit and vegetable worldwide besides potato, with more than 182 million tons produced worldwide on over 5 million hectares of cultivated area [12,13]. Meanwhile, several well-established industrial methods have been developed to improve the extraction efficiency of lycopene from tomatoes, making the tomato the primary source of lycopene [14,15,16]. 

Lycopene is an oil-soluble carotenoid with an aliphatic hydrocarbon chain with the molecular formula of C_40_H_56_ [17]. Lycopene has been demonstrated to be one of the most effective carotenoids for scavenging singlet oxygen and free radicals in vitro, as its molecular structure has 13 double bonds, including 11 conjugated double bonds that determine the antioxidant capacity of lycopene [18]. Epidemiological studies have also confirmed that lycopene is one of the most powerful antioxidants. Lycopene has twice the antioxidant strength of beta-carotene and ten times that of alpha-tocopherol. It has been found to be the most potent antioxidant carotenoid other than astaxanthin [19]. However, astaxanthin is an animal-derived antioxidant, and many vegetarians cannot accept it, so lycopene as a plant source is an excellent choice as a natural antioxidant. Due to the property of neutralizing reactive oxygen species, lycopene has been applied in cosmetics, such as incorporating lycopene in creams to treat melasma and protect the skin from ultraviolet radiation-induced erythema, matrix alterations, and mitochondrial DNA damage [20,21,22]. In addition, researchers have found that through antioxidant, anti-inflammatory, and antiproliferative activities, lycopene may exert preventive and therapeutic effects on different central nervous system illnesses, including Alzheimer’s disease, Parkinson’s disease, cerebral ischemia, epilepsy, and depression. In rodents with various clinical diseases, such as diabetes, colchicine exposure, high-fat diet, and aging, lycopene also enhances cognition and memory. In addition, lycopene protects against neurotoxicity induced by organic and inorganic pollutants, such as methylmercury, tert-butyl hydroperoxide, and cadmium [23,24,25]. This provides theoretical support for using lycopene in future clinical, therapeutic, and pharmaceutical applications.

Based on the above, the purpose of this review is to provide an overview of the specific forms of current applications of lycopene as an antioxidant in food products, and to provide a comprehensive summary of the effectiveness and progress of lycopene in the preservation of different types of food products over the last decade. The information in this review provides ideas for future applications of lycopene in food preservation as a natural antioxidant, which has great potential to replace traditional artificial antioxidants. 

## 2. Properties of Lycopene

### 2.1. Naturally Occurring Forms in Fruits and Vegetables

**Figure 1:**
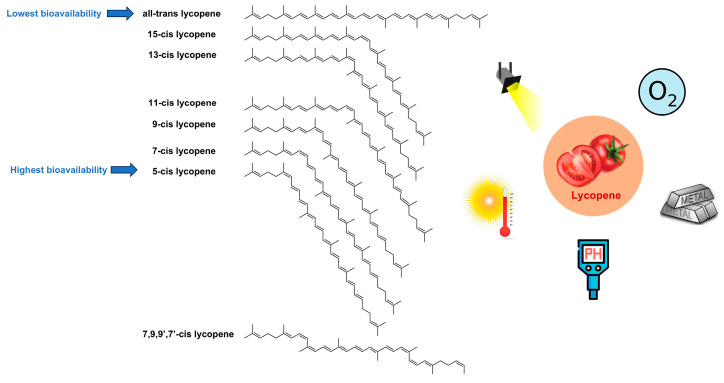
Different isomers of lycopene.

Lycopene is oil-soluble and found in many natural products, but mainly in red and orange fruits and vegetables, including tomato, pink grapefruit, papaya, pink guava, rosehip, apricot, watermelon, etc. [26]. Nevertheless, lycopene is also found in small amounts in some non-red and orange plants, such as asparagus and parsley [27]. Table 1 shows that the approximate content of lycopene in different vegetables and fruits varies significantly [20]. It is worth noting that in practical applications, different extraction methods and environmental factors (e.g., temperature, pH, light, time, etc.) can highly affect the actual amount of lycopene extracted [6,7,8]. For instance, Oberoi and Sogi [6] optimized the lycopene extraction from watermelon pulp using response surface methodology, and the extracted lycopene content ranged from 8.20 to 59.17 mg/100 g (on a fresh weight basis) as the independent variables were varied. Moreover, the lycopene naturally occurring in fruits and vegetables is predominantly all-trans isomers, while after processing, lycopene may be isomerized into different cis isomers, as shown in Figure 1 [28].

### 2.2. Bioavailability and Stability

Bioavailability refers to the relative amount of lycopene absorbed into the systemic circulation after oral administration, which is usually more important than the amount of active substance in the food because bioavailability directly reflects the proportion of the nutrient utilized by the body [29]. Several studies have shown that cis lycopene is more bioavailable than all-trans lycopene. Cooperstone et al. [30] reported that the bioavailability of lycopene in Tangerine tomatoes is about 8.5 times higher than in red tomatoes. This is most likely because 94% of the lycopene in Tangerine tomatoes is cis lycopene, whereas only 10% of the lycopene in red tomatoes is cis-structured. Other studies also have validated the above results. For example, when mice were orally administered a diet rich in cis lycopene, the total concentration of lycopene in the liver was more than 3-fold higher than that administered in a diet rich in all-trans isomer [31]. These results clearly indicate that cis lycopene exhibits greater bioavailability than the all-trans isomer. 

However, foods containing all-trans lycopene do not mean they cannot be absorbed and utilized by the body. After oral administration of all-trans lycopene into the stomach, it is usually isomerized to 5-cis, 9-cis, and 3-cis lycopene by gastric juice [32]. Among them, 5-cis lycopene is more bioavailable [33]. In the study of Richelle et al. [34], a significant (60%) proportion of this isomer was detected, while low levels of 9-cis and 13-cis lycopene were found in triglyceride-rich lipoproteins after eating a meal with corresponding lycopene isomers, suggesting that 9- and 13-cis isomers are mostly isomerized to 5-cis lycopene in the stomach or are less absorbed. 

Lycopene is extremely susceptible to oxidation, degradation, and isomerization due to the multiple conjugated double bonds in its chemical formula [35]. It is highly sensitive to external environmental factors, especially oxygen, heat, light, and metals (e.g., Cu^2+^ and Fe^3+^) [36]. Lycopene degrades more rapidly in dry heat environments, and its degradation rate accelerated with increasing temperature during heat treatment. While light has less effect on the loss of total lycopene, cis-isomer lycopene is less stable than all-trans lycopene during light exposure [37]. Oxygen is also an important factor affecting the stability of lycopene; the degradation rate of lycopene and dissolved oxygen consumption follow first-order kinetics, i.e., a linear relationship [38]. In addition, high-valent metal ions are reduced to low-valent metal ions or monomers mediated by lycopene, while lycopene is degraded during this period. This chemical reaction is more pronounced under acidic pH conditions, probably because metals are more soluble and reactive in acidic environments [39]. Therefore, controlling these factors during lycopene storage and processing is critical to maximize the efficacy of lycopene.

### 2.3. Benefits and Side Effects

Lycopene has several health benefits, notably reducing the incidence of various chronic diseases such as cardiovascular disease, oxidative stress, inflammation, cancer, and prostate disease [40,41]. As for side effects, moderate intake is negligible. However, excessive lycopene intake may cause health hazards, including lycopene dermatosis or lycopenemia. Lycopenemia results in yellowish–orange discoloration of the skin (usually seen on the palms, soles of the feet, and nasolabial folds) [42]. Although the symptoms are prominent, the condition itself is benign and harmless. After reducing lycopene intake, the patient’s condition improves significantly, and symptoms gradually resolve. 

There are no official recommendations for lycopene intake, but several studies have consistently shown that a daily intake of 2 to 75 milligrams of lycopene is safe [43,44]. Observational studies suggest that the dosage may potentially be increased to 270 mg/day without increasing the risk of adverse effects [45].

## 3. Forms of Lycopene Added to Foods

### 3.1. Direct Addition of Extracted Lycopene

Adding lycopene directly to food or during food preparation and processing is the most common method used nowadays to study lycopene for food preservation, because direct lycopene incorporation is easy and cost-effective for large-scale applications. The most primitive method is to add unrefined tomato products or by-products directly to food rather than to add extracted and refined lycopene crystals or lycopene oil. For example, Nour et al. [46] dried, cooled, and ground the waste (tomato skins and seeds) from a tomato processing plant to obtain a powder and add it to the bread dough. Similarly, tomato powder was added to sausages, i.e., Kim et al. [47] mixed tomato paste with olive oil, and dried and crushed them to obtain powder. The overall acceptability of the sausages with the addition of lycopene powder was much higher than that of the control sample. However, tomato waste is generally low in lycopene, with an average content of 174 mg/kg, as reported by Nour et al. [46]. When strong antioxidant properties are required in some products to extend shelf-life, large amounts of powder are added to increase the lycopene content, while the weight and volume of some food products are already limited, and large amounts of powder can seriously affect their color, volume, and weight to the detriment of their production.

To overcome this drawback, some researchers have used refined and extracted lycopene oils and crystals, as they have higher purity and usually only need to be added in small amounts to exert a powerful antioxidant effect. However, high-purity lycopene obtained by direct purchase is usually more expensive, as the extraction of lycopene requires a more complex process. For instance, Kehili et al. [48] used ethane to extract lycopene from tomato peel oleoresin at a concentration of 2%, which is approximately 100 times purer than the powder obtained from by-product processing. In addition to ethane, lipophilic organic solvents such as acetone and petroleum ether are widely used for lycopene extraction [49]. A rotary evaporator usually removes the solvent used for extraction to obtain a drier lycopene extract. Lycopene extract or refined tomato crystal is often used to preserve liquid oils, such as walnut oil, sunflower oil, and olive oil [48,50]. Also, high-fat products, such as anhydrous milk fat, butter, ice cream, and mayonnaise, can be enhanced with lycopene extract to extend shelf-life [51,52]. 

Unfortunately, the most significant limitation of direct lycopene addition is that, due to its oil solubility, it cannot be used in foods with high water content, such as fresh aquatic products, meat, and some unprocessed products. In this case, the method in the study by Ehsani et al. [53] immersed fresh foods in a solution containing lycopene for a period of time, followed by draining and packaging, can be considered a simple and effective way to extend the shelf-life of fresh products.

### 3.2. Composite Coating of Lycopene and Other Materials

Edible coatings are generally made from biodegradable materials and are based on various biopolymeric matrices, including polysaccharides, proteins, and lipids, which are non-toxic and non-harmful to humans. Edible coatings can be obtained by spraying onto the surface of food or immersing food directly into the solution, with the main purpose of extending the shelf-life of food and delaying spoilage [54,55]. This technique is commonly used to preserve fresh fruits, vegetables, and meat [56]. In addition, the combination of edible coatings and natural antioxidants has become a popular research topic in recent years to retard adverse flavor and color changes caused by the oxidation of lipids and other substances in foods, where natural antioxidants and antimicrobial agents are added to edible coatings to investigate their preservation effects [57]. 

Over the years, lycopene has been used as an emerging natural antioxidant for preparing composite coatings. According to a study by Ehsani et al. [58], a composite coating of sodium alginate and lycopene was applied to preserve rainbow trout. Sodium alginate is an excellent material for coating fabrication due to its biodegradability, eco-friendliness, sustainability, and elastic gel production in the presence of multivalent cations. Specifically, 2% (*w*/*v*) sodium alginate solution and 0.1% (*w*/*v*) calcium chloride solution were mixed to prepare alginate solutions with good clarity, and different levels of lycopene were dissolved in 10 mL 20% ethanol and 2.0 mL Tween 80 emulsifier. The mixed solution was added to the prepared alginate solution to form the coating solution. The samples coated with alginate containing lycopene showed significantly lower free fatty acid (FFA) and total volatile basic nitrogen (TVB-N) values compared to the control group. Interestingly, another study led by Ehsani et al. [59] three years later utilized a similar coating containing lycopene to further improve the coating based on this study. Unlike the previous research, the main component of the coating was chitosan. As in the previous study, lycopene was also incorporated into the coating solution to increase the antioxidant properties of the coating on top of that. Additionally, antibodies (IgY) isolated from egg yolk were added to the coating solution to further enhance the antimicrobial properties of the coating. A total of 2% (*w*/*v*) chitosan was dissolved in 1% (*v*/*v*) glacial acetic acid to make a chitosan solution. Then, the appropriate concentrations of lycopene were dissolved in 10 mL of 20% acetone, and 0.2% Tween 80 was added as an emulsifier to prepare a lycopene solution. The combination of lycopene and IgY used in coating showed a higher effect on delaying the rate of lipid oxidation. Moreover, composite coatings made from chitosan and lycopene were also used in the study by Khalida et al. [60] for grape preservation. 

### 3.3. Active Film Packaging with Lycopene

Nowadays, biodegradable films are a notable development in reactive packaging. On the one hand, edible film packaging has gained widespread popularity in recent years due to its natural composition, renewable nature, and environmentally friendly properties [61]. On the other hand, biodegradable films can be used as carriers of antimicrobial and antioxidant agents, including lycopene, to achieve the effect of controlling or preventing reactions that occur inside the packaging, thus extending the shelf-life of food products [62,63,64]. The main antioxidant mechanism of the packaging is the gradual migration of antioxidant substances from the film to the food substrate [65]. This leads to improved quality, longer shelf-life, control of pathogens, and improved sensory properties of food products. Therefore, active biodegradable film packaging has a wide range of prospects in the food industry. 

The most common material for biodegradable films is polylactic acid (PLA). PLA is a linear aliphatic thermoplastic polymer that can be produced from renewable energy sources. In a previous study [66], biodegradable films of lycopene-modified PLA were used in margarine packaging. The introduction of lycopene into the film reduces the oxidative reaction to improve the oxidative stability and color properties of the margarine. In addition, lycopene not only acts as an antioxidant, but also enhances the mechanical barrier and oxidation barrier of the thin film [67]. Adding red lycopene to biodegradable films helps reduce the films’ ultraviolet (UV) transmission, making them a good barrier to oxidation caused by UV radiation. In another study conducted by Stoll et al. [65], PLA films containing lycopene were subjected to release studies. According to a kinetic model, the lycopene in the films was gradually released into the food simulant with a release rate of up to 45% [65]. In general, larger molecules tend to have lower diffusion coefficients than smaller molecules, and compounds with larger structures should migrate at a slower rate. The higher flexibility of the lycopene chain reduces its intermolecular friction with PLA, facilitating the release of lycopene into the food simulant [65]. In addition to the direct addition of lycopene to films, Assis et al. [68] added lycopene nanocapsules to biodegradable films to prepare active packaging. The researchers measured a range of metrics in films spiked with different concentrations of free lycopene and lycopene nanocapsules. The results showed that the tensile strength and elongation of the films decreased when free lycopene was added. Similar results were also confirmed in the study by Tupuna-Yerovi et al. [69], where sodium alginate films containing lycopene showed lower tensile strength and elongation upon fracture. However, incorporating lycopene nanocapsule instead of free lycopene resulted in films with higher tensile strength and elongation, greater solubility, and the possibility to use this natural antioxidant [68]. Due to the oil solubility of lycopene, most of the studied bioactive packaging containing lycopene is now limited to its use in oily and fatty foods. Therefore, the combination of nanocapsules with biodegradable films could allow the films to have a wider range of applications in food packaging. The other thing to note is that cassava starch was used as the main material for making films in this study. Previous studies have shown that films made from cassava starch have good homogeneity, flexibility, transparency, and rapid biodegradability, and these properties also make it unnecessary to be processed before use [70]. This polymer, therefore, shows great promise for the development of films containing lycopene. Furthermore, in the production of antioxidant-active films, films made with only one film material alone often do not perform optimally. Based on this, Zeng et al. [71] developed a composite active film using sodium alginate and konjac glucomannan containing lycopene microcapsules to preserve sweet cherries and extend shelf-life. The films made from a mixture of two substances improved the limitations of sodium alginate or konjac glucomannan alone.

### 3.4. Emulsion Containing Lycopene

An emulsion is a system of two immiscible liquids, one of which is dispersed as droplets in the other, forming a heterogeneous dispersion system, which can be classified into water-in-oil (W/O) and oil-in-water (O/W) types. Emulsions are uniquely effective in improving the solubility, stability, and bioavailability of lipophilic compounds [72]. Since lycopene is fat-soluble, it is difficult to dissolve in water-based food systems, such as beverages, juices, and dairy products. O/W emulsions encapsulating lycopene in the oil phase not only improve its solubility in aqueous-based foods, but also prevent lycopene degradation in foods to some extent [73]. Due to its highly unsaturated hydrocarbon structure, lycopene is vulnerable to oxidative degradation by physical and chemical variables, such as light, oxygen, pH, and temperature during food processing, significantly reducing its biological activity and antioxidant capacity. Additionally, lycopene’s bioavailability and ability to release from the food matrix may be influenced by the structure and composition of the food. For fatty foods, the oxidation level is usually high. Fatty acids in the dispersed phase tend to deteriorate during long-term storage and reduce food quality. Emulsions encapsulated with lycopene can improve this situation, thus slowing down the oxidation of food products to achieve a longer shelf-life.

The most common emulsion is to dissolve lycopene directly in oil to form the oil phase, and then add the oil phase to the water phase to obtain a crude emulsion. In order to mix the water and oil phases and achieve the desired emulsion state, homogenization steps (e.g., using a high-pressure homogenizer) are generally required. In the study by Chen et al. [74], lycopene was added to corn oil to form an oil phase, and 5% (*w*/*w*) of the oil mixture was added to yogurt to improve the physical and chemical stability and rheological properties. To further improve the physical stability and bioavailability of lycopene beverage emulsions, Raikos et al. [75] designed a study to optimize the ratio of long-chain triglycerides (LCT) and short-chain triglycerides (SCT) in the oil phase. The results showed that the addition of LCT significantly reduced the average droplet size and enhanced the physical stability of the beverages by delaying the Ostwald ripening phenomenon. However, emulsions with high LCT content had higher creaminess and lower clarity than beverages with high SCT to LCT ratios, which may affect the sensory properties of the beverages. The most favorable formulation of beverage emulsions with lycopene is when the ratio of LCT (corn oil) to SCT (tributyrin) is 75:25.

Based on conventional emulsions, nanoemulsions have also been extensively studied and applied to food products in recent years. Nanoemulsions are prepared in a similar way to traditional emulsions, but the nanoemulsions are homogenized using an ultrasonic homogenizer to achieve an emulsion droplet size that is 10–100 times smaller particle size than ordinary emulsions [76]. In the study by Himanath et al. [77], nanoemulsions with coconut oil and soy lecithin were incorporated into yogurt as a stable delivery system for lycopene to improve antioxidant properties and maintain quality. It should be noticed that soy lecithin was used as an emulsifier in this study. Soy lecithin is the most commonly used food-grade phospholipid for the preparation of nanoemulsions and helps to improve the stability of nanoemulsions [78]. Good stability can ensure that the emulsion does not delaminate during long-time transportation and preservation, which extends the shelf-life of food.

Moreover, with the emergence of new technologies, nanotube technology has recently been applied to the encapsulation of lycopene. Chang et al. [79] encapsulated lycopene using α-lactalbumin nanotubes prepared by *Bacillus licheniformis* protease. Specifically, lycopene dissolved in ethyl acetate was added to the nanotubes, and the mixture was rotated on a rolling incubator to obtain α-lac nanotube emulsions containing lycopene. In (2,2-Diphenyl-1-picrylhydrazyl) DPPH radical scavenging experiments, the scavenging rate of lycopene-containing nanotubes was significantly higher than that of free lycopene when the lycopene concentration was higher than 10 μg/mL. Although the opposite results were obtained below 10 μg/mL, there was no significant difference when the statistical analysis was performed. In addition, the scavenging rate of lycopene nanotubes was much higher than that of lycopene in hydroxyl radical elimination experiments. Consequently, α-Lactalbumin nanotubes encapsulated with lycopene exhibited good antioxidant properties. 

### 3.5. Lycopene Microcapsules

Microencapsulation technology involves the encapsulation of liquids, solids, and even gases (core material) by selecting the appropriate film-forming material (wall material) to form small particles. The core material is encapsulated so that it is not in direct contact with the external environment and is effectively protected. Microencapsulation technology is now widely used in food to encapsulate unstable active ingredients, including lycopene, to protect and prevent oxidation, isomerization, and degradation during storage [80]. In addition, many micro- and nano-encapsulated bioactive compounds of plant origin can extend the shelf-life of foods due to their antimicrobial and antioxidant ability [81]. Academics are exploring the effects of lycopene microcapsules in food preservation to improve the antioxidant capacity of foods; lycopene microcapsules have now been successfully used in fresh-cut fruits to prevent browning and, thus, to extend the shelf-life [82].

Lycopene degrades in the presence of O_2_ and at high temperatures. Encapsulation may reduce these losses while allowing a controlled lycopene release over time. Previous studies have shown a less than 5% lycopene degradation rate for the obtained microspheres after 10 days at 10 °C [83]. Currently, many lycopene microcapsules have been successfully encapsulated using different wall materials, which play an active role in improving the stability of lycopene. Chen et al. [84] prepared lycopene microcapsules by a spray drying method using inulin, maltodextrin, and algal sugar, respectively, as wall materials and whey isolate protein–chitosan bilayer emulsion encapsulated with lycopene as core material. The embedding rate of microcapsules with inulin as the wall material was higher than that of microcapsules with alginate and maltodextrin as the wall material. The maximum encapsulation efficiency of 81.90 ± 1.20% was achieved when inulin was used as the wall material, and the core-to-wall mass ratio was 1:4. Meanwhile, Gheonea et al. [85] produced microcapsules by freeze-drying to minimize the oxidative degradation of lycopene during spray drying and to obtain a high-quality powder. Whey protein isolates and acacia gum were dissolved in distilled water at 2:1 (*w*/*w*) as wall material. Lycopene extract was dissolved in sunflower oil to be the core material. After homogenization, freeze-drying was carried out. The encapsulation efficiency of lycopene was 83.6 ± 0.20%. 

Moreover, several methods commonly used in food processing (e.g., Maillard reaction) have been used to modify the physicochemical properties of the microcapsule wall material to improve the protection of lycopene and flavor. Modified lycopene microencapsulation carriers effectively protect the microcapsules from harsh environmental influences that can lead to their destruction. The encapsulation efficiency and encapsulation yield were as high as 94% and 86%, respectively, when the weight ratio of whey protein isolate to xylo-oligosaccharide was 1:2, the reaction temperature was 90 °C, the heating time was 3 h, and the pH was 9.0. This is about a 12% improvement in encapsulation efficiency compared to the previously mentioned encapsulation materials of whey protein isolate and acacia gum. Also, glycosylation of whey protein isolate significantly improved the emulsification properties and antioxidant capacity of lycopene. The protective effect of glycosylated whey protein isolate on lycopene was superior to that of whey protein isolate alone [86]. There are also novel wall materials that have been used to encapsulate lycopene. Pu et al. [87] improved the stability of lycopene by encapsulating it in *Chlorella* cells. After storage at 25 °C, the complex maintained a higher antioxidant effect than free lycopene. Souza et al. [88] investigated the effect of maltodextrin, whey protein isolate, and octenyl succinic anhydride (OSA) modified starch on the stability of lycopene in microcapsules. The results showed that maltodextrin and OSA-modified starch provided stronger lycopene protection and greater antioxidant capacity. Compared to unencapsulated lycopene, lycopene microcapsules had 2.0 to 2.4 times greater free radical scavenging capacity. 

## 4. Effect of Lycopene as a Natural Antioxidant on the Preservation of Different Types of Foods

**Figure 2:**
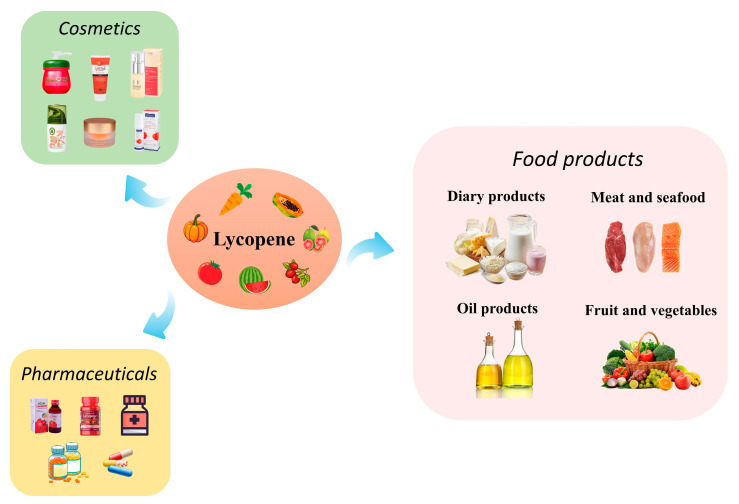
Applications of lycopene in different fields.

Lycopene, as a natural antioxidant, has a wide range of applications in cosmetics, pharmaceuticals, and food products (Figure 2). In the food field, lycopene is successfully used in meat, seafood, oil products, dairy products, fruits, vegetables, etc., showing good results in preserving freshness and extending the shelf-life of foods; more details are provided below.

### 4.1. Meat and Seafood

Lycopene is currently used in the preservation of aquatic products in many ways. However, lycopene is introduced in meat products mainly through the addition of processed tomato products, such as tomato paste and tomato powder [47,89].

Kim et al. added tomato powder formulated at 0% (C), 0.8% (T1), 1.2% (T2), and 1.5% (T3) levels to the basic recipe of low-fat pork sausage [47]. Thiobarbituric acid reactive substances (TBARS) values were used to measure the level of lipid oxidation in the sausages. After 15 days of storage, the team observed a significant decrease in TBARS values in the T2 and T3 groups (*p* < 0.05); however, no significant changes were observed in the C and T1 groups (*p* > 0.05). Thus, the low-fat pork sausage added with tomato powder showed lower lipid oxidation due to the existence of lycopene in tomato products. Similar results were obtained in a study where tomato paste was added to pork burgers [89]. Based on this, processed tomato products can be a source of lycopene as a functional additive and natural antioxidant in human diets. 

Lycopene has been shown to have a more pronounced effect on the preservation of fish. The freshness or nutritional value of fish is evaluated by measuring oxidation indicators, such as FFA, peroxide value (PV), TVB-N, and thiobarbituric acid (TBA), during storage according to existing methods [90,91,92]. The effect of alginate coating without or with lycopene on the quality of rainbow trout stored under refrigerated conditions for 16 days was investigated by Ehsani et al. [58] in 2017. Rainbow fish samples coated with alginate showed significantly lower FFA and TVB-N values than the control group. Furthermore, significant differences between these two values were observed between coated samples with and without lycopene at the end of storage. It is recommended that alginate coating with a 3% lycopene level be applied to rainbow trout preservation, as the samples of this combination had the best performance in the study. This team has been working hard on using lycopene to preserve rainbow trout in recent years, and in 2020 they went even further to improve the edible coating containing lycopene. The group led by Ehsani studied the effect of chitosan-based coating containing extracted egg yolk antibodies and lycopene on rainbow trout fillets’ chemical quality during 16 days of refrigerator storage [59]. Results showed that chitosan solutions with lycopene or IgY could significantly (*p* < 0.05) increase the oxidative stability of lipids in fish fillets with lower FFA, PV, and TBA values. Moreover, the combinational use of lycopene and IgY showed a higher effect on delaying the rate of lipid oxidation compared to adding one separately. Thus, adding egg yolk antibodies and lycopene to the coating solution reduces lipid oxidation, making them good bio-preservatives for seafood products. Interestingly, the investigators in this study introduced pH to evaluate the freshness of the fish, and pH levels above 7.1 can be considered an indicator of spoilage. As a result, the control group had a pH value above 7.1 on the 12th day, indicating impaired quality of fish, while no significant changes were found in the other treatments during the 16-day storage period.

The above measurements are theoretical indicators of fish during storage, but they are not representative of consumers’ acceptability of them when they purchase and consume the product. Therefore, it would be an excellent choice to include sensory evaluation to assess the effect of lycopene on fish in storage, which would provide a more intuitive response to the quality and freshness of fish and meat. In the sensory evaluation of meat, panelists usually evaluate color, aroma, flavor, tenderness, juiciness, and overall acceptability. The results of the study by Kim et al. showed that the scores for color, aroma, tenderness, and juiciness were mostly comparable to the control or sometimes even better, but without significant differences (*p* < 0.05) [47]. The values of flavor were much higher than samples without tomato powder (*p* > 0.05). For the fish, color, odor, texture and general acceptability were rated by the evaluation group. In another study conducted by Ehsani et al. [53], the team took two different approaches to introducing lycopene into rainbow trout. The research team first fed sample rainbow trout to dry pellet diets containing different concentrations of lycopene. Subsequently, at the end of the feeding trial, 30 fish, randomly selected from each treatment, were immersed in different concentrations of lycopene (*w*/*v*) solutions. The results showed that lycopene (endogenous and exogenous) was very effective in stabilizing the freshness of trout fillets during refrigeration (*p* < 0.05). The effect of exogenous lycopene on PV, TBA, FFA, and sensory scores was more significant compared to endogenous lycopene and the control group. For the sensory evaluation scores, the fillet samples receiving dietary lycopene reached the limits of acceptability for odor and texture after 12 days, while the samples receiving exogenous lycopene never reached this limit during the entire storage period. Based on this, exogenous lycopene, especially higher levels of lycopene, may be the most effective means of extending the shelf-life of trout fillets during storage. 

### 4.2. Oil Products

Lipid oxidation may occur in edible oils at various stages, from production, storage, and purchase to consumption, leading to major health issues, such as heart disease, mutagenesis, and carcinogenesis. Oxidative deterioration is one of the most important factors limiting the shelf-life of oil products, which increases the formation of toxic aldehydes through the degradation of polyunsaturated fatty acids and reduces the nutritional value of foods. Lipid oxidation also changes food sensory properties, such as poor flavor, color, and texture. How to reduce and retard lipid oxidation has been a critical issue that needs to be addressed to extend the shelf-life of oils. Lycopene has had remarkable success in the preservation of oils. 

In China, most barrel oils are usually supplemented with relatively inexpensive synthetic antioxidants such as TBHQ, BHA, and butylated hydroxytoluene (BHT) to extend their shelf-life [93]. Whether natural antioxidants can have the same or even better antioxidant effects than synthetic antioxidants has become an important research topic. In recent years, researchers have compared the effectiveness of lycopene and synthetic oxidants in preserving oil from different perspectives. Xie et al. [50] observed that 0.005% lycopene level was superior to the common antioxidants BHA and BHT in inhibiting the increase in the acid value of walnut oil. Also, the greatest anti-DPPH activity of BHT in the research by Varas Kehili et al. [48] remained stable at roughly 65% for BHT concentrations between 25 and 200 g/mL, but that of lycopene extract reached a value of 98% at a lycopene concentration of 200 g/mL. In another study on the effect of the shelf-life of lycopene linseed oil, almost the same oxidative stability index was obtained in the presence of 80 mg lycopene/kg linseed oil and using 200 mg/kg artificial antioxidant BHT at 110 °C [94]. 

Due to the oil solubility of lycopene, the main way lycopene is used in oil preservation is by direct addition of lycopene extract or tomato processing by-products. Xie et al. [50] evaluated the effect of lycopene on the quality of walnut oil by measuring the PV, acid value, fatty acids, total phenolic content, and ferric-reducing antioxidant power (FRAP) of walnut oil. When lycopene was added directly to the oil, higher concentrations did not mean a better preservation effect. After 45 days of accelerated oxidation at 60 °C (Schaal oven test), the addition of 0.005% lycopene exhibited the greatest antioxidant effect over other lycopene levels. The 0.005% lycopene had significantly lower PV than the 0.010 and 0.015% lycopene (*p* < 0.05), suggesting that higher lycopene concentrations may not improve PV. This may be because higher concentrations of lycopene may interact with other minor components, such as phenolic compounds, phytosterols, squalene, and fatty acids, thus antagonizing the antioxidants [95]. In addition, the saturated fatty acids, unsaturated fatty acids, total phenolic content, and FRAP values of polar and non-polar components were significantly higher in walnut oil supplemented with 0.005% lycopene compared to the blank sample (walnut oil without any lycopene addition). Among them, higher FRAP values represent higher antioxidant capacity [96]. Under normal environmental storage conditions, the previous shelf-life of walnut oil of approximately 2 months could be extended to 16 months by adding 0.005% lycopene. 

The recommended levels of lycopene addition vary in different types of oils. A 0.005% lycopene level is recommended for the preservation of walnut oil, but the recommended concentrations in olive oil and sunflower oil are quite different. Kehili et al. [48] utilized lycopene-rich oleoresin extracts (TPO) from tomato industrial by-products as an effective natural antioxidant alternative to synthetic preservatives in refined olive oil (ROO) and refined sunflower oil (RSO), thereby slowing down oxidation reactions during long-term storage. Different TPO doses exhibited different antioxidant activity levels in ROO and RSO. This study recommends the use of 250 µg/g and 2000 µg/g TPO containing 5 µg/g (0.0005%) and 40 µg/g (0.004%) lycopene for the preservation of ROO and RSO, respectively. High-concentration TPO promoted oxidative reactions in ROO samples during long-term storage, while no such effect was found in RSO samples. Thus, the protective effect of TPO on primary oxidative reaction damage in different oils was highly correlated with their lycopene content. 

The effect of carotenoids on lipid oxidation is concentration dependent. Considering that carotenoids inhibit lipid oxidation mainly by capturing peroxyl radicals, their antioxidant mechanism is closely related to the oxidation of carotenoids themselves [97]. However, the autoxidation of carotenoids generates a complex mixture of products with epoxides, hydroxyl groups, and carbonyl groups, which may enhance the propagation phase of the oxidation reaction and further induce oxidative deterioration by providing the system with more oxidizable substrates [98]. To overcome the disadvantages of adding lycopene directly to the oil, more and more researchers have started to design suitable packaging techniques for oil preservation in recent years. Pirsa et al. [66] developed PLA/titanium dioxide (TiO_2_)/lycopene (Lyc) nanocomposite films for the packaging of margarine. The oxidative properties of margarine (antioxidant activity, acidity value, PV, and TBA value) showed that the quality of margarine decreases during storage, but the PLA/TiO_2_/Lyc films controlled the oxidation factor and significantly extended the shelf-life of margarine (*p* < 0.05). PLA films containing lycopene were also used to preserve sunflower oil by Stoll et al. [65]. When sunflower oil was exposed to light, the film containing lycopene protected the oil by its oxygen barrier and lycopene release, effectively scavenging free radicals formed during all stages of lipid oxidation. The results of several studies have shown that films containing lycopene are very effective in preserving the oil. 

### 4.3. Dairy Products

Milk is considered a nutritious food because it is fresh and can be stored for long periods. In some countries, almost half of all milk is consumed as fresh, pasteurized whole, low-fat, or skimmed milk. However, most milk is manufactured into more stable dairy products that are commercialized globally. Dairy products refer to milk and any food made from milk, and are extremely diverse, including butter, cheese, ice cream, yogurt, condensed milk, and dried milk. Among the dairy products, the most perishable are those with high-fat content, such as cream and butter. Oxidative lipid rancidity is a major problem that occurs during the processing and storage of high-fat dairy products [99]. It affects many characteristics of dairy products, such as color, flavor, texture, and nutritional value. Based on this, several articles have reported that adding lycopene to dairy products can slow down lipid oxidation.

For instance, Kaur et al. [52] incorporated lycopene crystals into butter (20 ppm) and ice cream (70 ppm) and analyzed them for storage stability indices and sensory characteristics during storage for 4 months. The results showed that the control butter samples showed a significant increase in PV after 2 months of storage (*p* < 0.05), while the experimental butter containing lycopene showed a significant increase in PV only after 3 and 4 months. For ice cream, both the control samples and the samples with lycopene showed a significant increase in PV with increasing storage time (*p* < 0.05), but it also showed a slow increase in PV for the ice cream containing lycopene. Similar results were seen in the variation of FFA, i.e., both the butter and ice cream samples containing lycopene had significantly lower FFA content than the control samples at the end of storage. Siwach et al. [51] also reported that the addition of lycopene at all levels from 30 ppm to 150 ppm (*p* < 0.05) significantly reduced the development of rancidity. Lycopene contributed significantly (*p* < 0.05) to the change in FFA content in the anhydrous milk fat, and samples containing lycopene exhibited significantly lower PV throughout the storage period compared to the control anhydrous milk fat (*p* < 0.05). Notably, the higher the concentration of lycopene, the higher the antioxidant effect observed in terms of peroxide inhibition. Moreover, TBA was also considered to detect fatty food oxidation deterioration and to measure lipid oxidation secondary products [100]. Significantly lower (*p* < 0.05) TBA values were observed in the anhydrous milk fat sample containing lycopene compared to the control sample.

In addition to measuring chemical indicators, sensory characteristics are also one of the most important indicators of the acceptability of dairy products to detect sourness and spoilage. In general, sensory panel members rate dairy products for sensory attributes, such as odor, taste, texture, and color, throughout their storage period. For example, Ehsani et al. [99] observed that the scores of pasteurized cream containing lycopene decreased less compared to the control group, and all scores were accepted by the panelists during storage. The taste scores of the samples containing lycopene decreased more slowly during storage than the other treated creams and the control. A similar sensory evaluation was also conducted in the study of Siwach et al. [51]. Anhydrous milk fat scored significantly higher than the control in terms of flavor at the end of 8 and 12 months of storage because lycopene was effective in preventing oxidative rancidity. Moreover, the flavor was compared between lycopene added at 150 ppm and anhydrous milk fat added with BHA at 200 ppm, and no significant difference was found. However, the addition of 150 ppm level (the highest level) of lycopene to the samples received the lowest score in terms of color, due to the unacceptable dark red color resulting from the high levels of lycopene. This phenomenon also reminds researchers and manufacturers that the effect of lycopene on the color of the buffalo milk fat is a limiting factor for the use of concentrations above 150 ppm. A previous study [52] also claimed a significantly later reduction in sensory scores in cream and ice cream with lycopene than in the control group. In addition to the color effect, the above findings suggest that lycopene has great potential as an antioxidant in extending the shelf-life of dairy products.

For liquid milk such as yogurt and milk, which have a lower fat content compared to butter and cream, the direct addition of lycopene may lead to layer separation, so researchers usually prepare emulsion containing lycopene first and then add the emulsion to dairy products [74,77]. According to the results of the storage stability experiment, the incorporation of lycopene nanoemulsions not only did not have any effect on the physicochemical properties of the yogurt, but also enhanced the antioxidant activity and maintained the quality of yogurt for 28 days [77]. It also helped to improve the viability of *L. delbrueckii subsp. bulgaricus* in the yogurt, which also improved its quality retention. In addition, yogurt containing lycopene nanoemulsions had more acceptable organoleptic properties compared to plain yogurt samples. An interesting phenomenon found by Chen et al. [74] was that the PV of yogurt containing lycopene emulsion remained relatively stable and only decreased slightly even after 21 days of storage (*p* > 0.05). Furthermore, the water retention capacity of yogurt containing lycopene emulsions increased significantly with storage time. 

### 4.4. Fruits and Vegetables

Fresh-cut fruits are favored by people due to their health, high nutrition, convenience, good flavor, etc. However, fresh-cut fruits deteriorate faster than unprocessed fruits, mainly due to the damage caused by processing methods (e.g., peeling, slicing, chopping). These processing operations usually shorten the shelf-life of fresh-cut fruits and vegetables, such as tissue softening, browning of cut surfaces, loss of nutritional value, presence of off-flavors, and microbial deterioration during storage [101,102]. Fresh-cut fruit remains a challenge for preservation, mainly due to the browning changes that occur when the membranes inside the cells are disrupted after the fruit has been cut and exposed to air. Since color changes make it difficult for consumers to accept and purchase, research into fresh fruit preservation methods is of considerable importance for commercial trade. Recently, applying natural antioxidant substances to fresh fruit preservation and adding antioxidants and antimicrobial substances to packaging to prepare active packaging materials have been hot research topics [103]. 

Since fresh fruit can be consumed directly, adding lycopene extracts or crystals during the preparation process, like other food products, is not practical. Therefore, a common treatment method used in academia today is to coat the surface of fruits. Zeng et al. [71] developed composite films containing lycopene microcapsules and investigated the effect of prefabricated composite films on the preservation of sweet cherries stored at 0 °C for 15 days. The results showed that the composite films effectively extended the shelf-life of sweet cherries and delayed the decline in decay rate, pH, soluble solids content, and other indicators. It also contained the powerful antioxidant lycopene, which reduced the oxidation of vitamin C and removed free radicals from cherries, thereby extending their shelf-life and maintaining their nutritional value during storage [71]. Other teams have used chitosan-based coatings or films containing lycopene to preserve fresh fruit. For example, a study on the effect of edible coatings containing lycopene on the ability to preserve grapes has been conducted [60]. The results showed that the combination of 1.5% (75 lycopene: 25 chitosan) had the greatest preservation capacity compared to the other experimental groups, with a 2-fold slower mass degradation and a 2-fold slower decrease in water content than the control. 

The method of soaking in a solution containing lycopene is also used for the preservation of fresh-cut fruits. A research group macerated fresh-cut apples in a solution containing lycopene microspheres to improve their freshness and nutritional value during preservation [82]. Enzymatic browning of freshly cut apples immersed in a solution of lycopene microspheres was controlled after 9 days at 5 °C, which is most likely due to the high antioxidant properties of lycopene. Lycopene can chelate oxygen with a chelation constant twice as high as that of β-carotene [104]. The release of lycopene from the microspheres highly controls the enzymatic oxidation of phenolic compounds. The results also showed that this method did not affect the physicochemical or microbiological quality of fresh-cut fruits. Furthermore, after 9 days at 5 °C, the incorporation of lycopene microspheres increased the health function of fresh-cut apples and increased phenolic compounds by up to 56% (chlorogenic acid). 

In summary, the applications of lycopene as an antioxidant in different types of foods are listed in Table 2.

## 5. Conclusions

The outstanding anti-inflammatory, antioxidant, and anti-tumor properties of lycopene have made it one of the potential pharmaceutical ingredients, especially for treating and preventing cardiovascular diseases, prostate cancer, and diabetes. Meanwhile, lycopene has also become an emerging active ingredient in the cosmetic field for anti-aging because of its powerful antioxidant activity that protects against free radical damage to the skin. In addition, lycopene has many advantages in food preservation, such as increasing the health and antioxidant properties of food, or adding lycopene to packaging can have good barrier properties to extend the shelf-life of foods, thus improving the quality of foods during storage. 

However, lycopene is sensitive to external environmental factors such as light, heat, oxygen, and metal ions. The degradation of lycopene is accelerated when induced by these environmental factors. In addition, the oil solubility of free lycopene has led to strict limitations on the types of foods. Currently, the direct addition of lycopene to foods is limited to oils such as vegetable oils, butter, and anhydrous cream. If added directly to foods in aqueous systems (e.g., beverages, yogurt, and fresh aquatic products), the antioxidant effect of lycopene will be affected because of the layering phenomenon. Meanwhile, researchers usually report that lycopene affects color scores in the sensory evaluation of foods in studies that have been conducted. This may be attributed to the inherent color properties of lycopene, which is dark red at high concentrations (>100 ppm). Other studies have claimed that adding free lycopene directly to the film reduces its ductility and tensile strength, making the film susceptible to breaking.

To overcome the above limitations, researchers have introduced various ways (e.g., edible coatings, biodegradable films, emulsions, and microcapsules) to combine them with lycopene, showing better application prospects and results. Novel carriers not only prevent oxidation, isomerization, and degradation of lycopene during processing and storage, but also ameliorate the limitations of lycopene application, the negative impact on food sensory, and the properties of films. On the basis of loading lycopene on suitable carriers, much of the current cutting-edge research is combining lycopene with antimicrobial substances or other active substances for preservation, such as antibodies, TiO_2_ particles, chitosan, sodium carboxymethylcellulose, etc. Therefore, combining lycopene with other active substances will be a major trend in the future, so that one plus one can achieve a more significant effect than two. 

## Figures and Tables

**Table 1:** Lycopene content in different natural sources.

Food Sources	Contents (mg/100 g)
Tomato	0.72–4.2
Pumpkin	0.38–0.46
Sweet potato	0.02–0.11
Pink grapefruit	0.35–3.36
Carrot	0.65–0.78
Pink guava	5.23–5.5
Watermelon	2.30–7.20
Apricot	0.01–0.05
Papaya	0.11–5.3
Rosehip	0.68–0.71

**Table 2:** Lycopene is added as an antioxidant in different food products.

Food Products	Lycopene Addition Form	Concentration of Lycopene	Results	Refs.
Low-fat pork sausage	Tomato powder	No mention	Tomato powder up to 1.5% was found to be well acceptable for up to 30 days at refrigerated storage.	[47]
Pork burgers	Tomato paste	145.5 ± 8.8 mg/100 g	Tomato paste containing lycopene increased the lipid oxidation stability of burgers during storage.	[89]
Rainbow trout	Alginate coating with lycopene	0.5, 1, 1.5, and 3% *w*/*v*	Alginate coating with 3% lycopene had the best performance.	[58]
Rainbow trout	Chitosan-based coating containing extracted egg yolk antibody along with lycopene	1.5 and 3%	Egg yolk antibodies and lycopene to the coating solution significantly reduced lipid oxidation and improved sensory attributes.	[59]
Walnut oil	Lycopene with a purity of 95%	0.001 to 0.015%	0.005% lycopene exhibited the greatest antioxidant effect.	[50]
Linseed oil	Lycopene extract from tomato	40, 80 and 120 mg lycopene/kg oil	The oxidative stability index of 80 mg lycopene/kg in linseed oil at 110 °C is the same as that of 200 mg/kg BHT.	[94]
Refined olive and sunflower oils	Lycopene-rich oleoresin extracts	5, 10, 20, and 40 µg/g	It is recommended that refined olive oil and sunflower oil be preserved with 5 µg/g and 40 µg/g lycopene, respectively.	[48]
Margarine	Polylactic acid /titanium dioxide/lycopene nanocomposite film	3% *w*/*v*	The film controlled the oxidation factor and significantly extended the shelf-life of margarine.	[66]
Sunflower oil	Polylactic acid films containing lycopene	No mention	The oxygen barrier and lycopene release properties of films improve the oxidative stability of sunflower oil.	[65]
Butter and ice cream	Lycopene crystals	Butter (20 ppm) and ice cream (70 ppm)	Lycopene improved the oxidative stability of butter and ice cream during 4-month storage.	[52]
Anhydrous cow milk fat	Lycopene extract	30, 60, 90, 120, and 150 ppm	Lycopene at all levels significantly prevented the development of oxidative rancidity in milk fat	[51]
Pasteurized cream	Lycopene extract	20 and 50 ppm	Lycopene prolonged the shelf-life of the cream, and the results of the sensory evaluation were acceptable during storage.	[99]
Yogurt	Emulsion	1.5 mg/g oil phase	Improved physicochemical stability and rheological properties after the addition of lycopene to yogurt using emulsion.	[74]
Yogurt	Nanoemulsion	0.2, 0.4, 0.6, 0.8, and 1%	Yogurt containing lycopene nanoemulsions showed better antioxidant and physicochemical properties, as well as improved survival of *Lactobacillus bulgaricus* for up to 28 days.	[77]
Sweet cherry	Composite film containing lycopene microcapsules	10 mL lycopene extract oil to prepare microcapsules	Lycopene effectively reduced cherry oxidation and extended shelf-life during storage.	[71]
Grape	Edible coating containing lycopene	0.375, 0.75, 1.125, and 1.5%	The combination of 1.5% (75 lycopene: 25 chitosan) had the greatest preservation capacity.	[60]
Fresh-cut apple	Lycopene microspheres	5 mL cis–lycopene-rich oil was added to 50 mL of gelatine solution to form a microsphere	The dipping treatment of fresh–cut apples, including 2 g/L lycopene microspheres, reduced browning while quality was maintained, and some bioactive compounds even enhanced after 9 d at 5 °C.	[82]

## Data Availability

Data included in this study are available on request.
